# Clinical Epidemiology of Malaria in the Highlands of Western Kenya

**DOI:** 10.3201/eid0806.010309

**Published:** 2002-06

**Authors:** Simon I. Hay, Abdisalan M. Noor, Milka Simba, Millie Busolo, Helen L. Guyatt, Sam A. Ochola, Robert W. Snow

**Affiliations:** * University of Oxford, Oxford, UK; † Kenya Medical Research Institute/Wellcome Trust Collaborative Programme, Nairobi, Kenya; ‡ Ministry of Health, Nairobi, Kenya

## Abstract

Malaria in the highlands of Kenya is traditionally regarded as unstable and limited by low temperature. Brief warm periods may facilitate malaria transmission and are therefore able to generate epidemic conditions in immunologically naive human populations living at high altitudes. The adult:child ratio (ACR) of malaria admissions is a simple tool we have used to assess the degree of functional immunity in the catchment population of a health facility. Examples of ACR are collected from inpatient admission data at facilities with a range of malaria endemicities in Kenya. Two decades of inpatient malaria admission data from three health facilities in a high-altitude area of western Kenya do not support the canonical view of unstable transmission. The malaria of the region is best described as seasonal and meso-endemic. We discuss the implications for malaria control options in the Kenyan highlands.

The temperate highlands of western Kenya were regarded by colonial settlers as safe havens from the surrounding malarious areas of Uganda and Kenya (*1*,*2*). After World War I, malaria encroached into these highland communities as a result of wide-scale population settlement linked to transport and agricultural development (*2*–*6*), and malaria epidemics were frequently reported by the early 1930s (*7*–*11*). These epidemics in the highlands caused concern to those in the colonial administration because of the economic importance of agricultural exports. During the 1950s and 1960s, control efforts such as indoor residual house-spraying, mass drug administration, or chemoprophylaxis effectively contained or prevented epidemics in some of these high-altitude areas (*12*–*15*).

In the late 1980s and early 1990s, a series of malaria “epidemics” were reported in Kenya and other communities located at high altitudes in the subregion (*11*,*16*–*26*). Some authors have labeled these resurgences as a new typology variant, “highland malaria,” demanding special attention in the new global commitment to Roll Back Malaria (*27*–*29*). A generally accepted view has been that the transmission of *Plasmodium falciparum* in high-altitude communities is limited by low ambient temperature. Small changes in climate may therefore provide transiently suitable conditions for unstable transmission in populations that have acquired little functional immunity (*8*,*9*,*30*).

The highlands of Kenya constitute a densely populated, politically significant area, which serves as a major source of revenue and foreign exchange from agricultural exports. The Kenyan government has recently defined 15 districts in the highlands (*31*,*32*) as being prone to epidemics, meriting close inspection, preparation, and intervention (*33*). We examine a time series of age-structured clinical malaria data derived from three hospitals with inpatient admission facilities in the highlands of western Kenya. These data provide an empirical basis for understanding the epidemiology of malaria and consequent strategic approaches to disease management and prevention in this area. A companion paper investigates the epidemiologic and statistical problems associated with defining true epidemics in these high-altitude locations and tests a variety of epidemic surveillance algorithms on the monthly malaria admissions series abstracted from these facilities (*32*).

## Methods

The location of the three hospitals that provided inpatient clinical care and were identified for use in this study, along with details about the collection of clinical data and the local weather conditions, are provided in our companion paper (*32*).

Providing a precise catchment area of the population for admission’s data was not possible as such information is not routinely collected in the hospitals; we assumed therefore that most inpatients came from the immediate surrounding high-elevation catchment area. Typically, long-term, facility-based data are difficult to interpret without some estimate of the populations served and how the population may have changed over time. To provide demographic information on the number of people served by the hospitals, we used population estimates from Kenyan national censuses in 1979 (*34*), 1989 (*35*), and 1999 (*36*). District and lower level administrative boundaries changed with each census, so population growth rates were defined for three contiguous administrative areas, as close to the hospital as possible, which had not been subjected to boundary redefinition. Intercensal population growth rates (r) were calculated by using the formula r=log_e_(t_2_–t_1_), where t_1_ is the population estimate of the first census and t_2_ the population estimate of the second.

Data manipulation and statistical transformation were performed in Excel 2000 (Microsoft Corp., Seattle, WA) unless otherwise stated. Monthly mean adult (>15 years) and child (<15 years) admissions were calculated and displayed as spider plots. The time series of admissions at each site was also plotted with a 25-point (month) moving average of the series to show more clearly the long-term movement in these data. For each hospital, we performed trend analysis through linear regression of the malaria admission data against a trend variable (observation month number/12). The coefficient of trend therefore indicates the annual trend (positive = increasing in time, negative = decreasing in time, zero = a stationary series). Such regression models are sensitive to seasonal variation, outliers, and heteroscedasticity (a term which refers to situations in which the variability of the residuals is not constant). To show the long-term trend unambiguously, seasonality was removed from each series by using an additive seasonal decomposition procedure (*37*,*38*) and the residuals were checked for normality and heteroscedasticity in SPSS version 11 (SPSS Inc., Chicago, IL).

Furthermore, we applied a proximate measure of transmission stability through a comparison of the numbers of adults to children with malaria admitted to the three hospitals. This adult:child ratio (ACR) of cases was calculated from total adult and child admissions for the duration of the available records. In areas of stable transmission, we assumed that the risks for complicated malaria in adulthood would be significantly lower than the risks in childhood; this assumption was based on expectations of the age distribution of malaria cases under varying transmission intensities (*39*–*41*). Conversely, areas of infrequent parasite exposure lend themselves to equivalent risks in adults and children. As such, an ACR derived from hospital admissions approaching unity would suggest an increasing tendency toward unstable transmission, assuming that the typical age-structured population pyramid for developing country and rural communities prevailed (*36*) and that there were no age-dependent biases in attendance rates.

## Results

**Figure 1:**
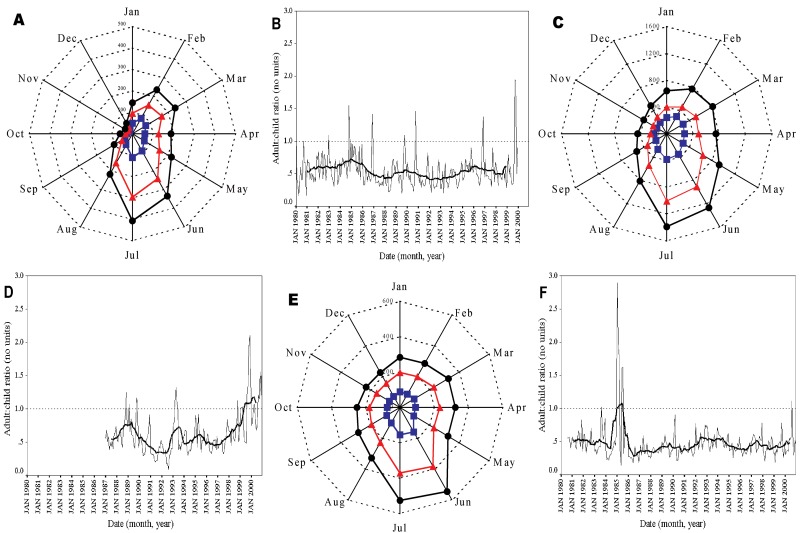
Spider plots of adult, child, and total admissions and time series of adult:child ratio for three study hospitals in Kenya. Spider plots of malaria admissions in Kilgoris (a), Kisii (c), and Tabaka (e). The data are monthly averages for the 1980–1999, 1987–2000, and 1981–2000 time periods, respectively. Adult cases (>15 years of age) are shown in blue, child cases (<15 years) are shown in red, and total cases in black. Time series plots of the monthly adult:child ratio data are also shown for Kilgoris (b), Kisii (d), and Tabaka (f) as the continuous black line. The dashed line represents the value of 1 where adult and child admissions are equal, as is to be expected in true epidemic conditions (39–41). The bold line is a 25-point (month) moving average of the adult:child ratio.

Time series of malaria admissions from 1980 to 1999, 1987 to 2000, and 1981 to 2000 were recorded for Kilgoris, Kisii, and Tabaka hospitals, respectively. These clinical data represent 171,312 admissions with a primary, coprimary, or coincidental diagnosis of complicated malaria over a total of 54 admission years. The Kilgoris, Kisii, and Tabaka hospitals managed an average of 2,243; 9,191; and 3,929 malaria admissions per year, respectively, for the duration over which records were available (Table 1). Throughout the study period, the frequency of childhood admissions was on average twice that of adult admissions (Table 1; Figure 1a,c,e). The average ACR calculated for all months was 0.46 for Kilgoris (14,079/30,793), 0.52 for Kisii (44,043/84,648), and 0.42 for Tabaka (23,692/55,871). The ACRs derived from monthly admissions are relatively constant throughout the duration of observation (Figure 1b,d, f). Several anomalies in these data were evident, however, particularly at Tabaka in 1985 and Kisii in 1999; we did not identify any obvious explanation for these exceptions in the time series, although they did not occur during periods of major epidemics at the sites (*32*). In further analysis, we focused on the primary pediatric clinical case data. We considered data from children to be more likely to give an accurate picture of local malaria transmission, as they are less likely to have developed functional immunity or to have traveled and acquired infections elsewhere.

**Table 1:** Mean monthly child and adult admissions at the three study hospitals, Kenya^a^

	Mean monthly admissions												
	Jan	Feb	Mar	Apr	May	Jun	Jul	Aug	Sep	Oct	Nov	Dec	Total
Kilgoris (1980-1999) Child	96	154	164	127	151	242	296	158	60	34	27	34	1,543
Adult	49	81	73	61	68	94	112	60	41	26	16	21	702
Kisii (1987–2000)													
Child	401	465	515	483	632	913	1,000	539	336	248	233	281	6,046
Adult	239	300	285	267	285	357	387	271	193	190	173	200	3,147
Tabaka (1981–2000)													
Child	197	201	229	231	231	385	371	230	193	178	158	156	2,760
Adult	87	85	97	91	93	160	154	101	87	74	69	71	1,169

^a^Children are defined as <15 years of age. Adults are >15 years.

The long-term data used in this analysis indicate that clinical cases of malaria occur every month at each hospital; acute seasonal peaks occur in June and July (Figure 1a,c,e). On average, one third of the total annual child malaria admissions were concentrated in these 2 months (35%, 32%, and 27% for Kilgoris, Kisii, and Tabaka, respectively).

**Figure 2:**
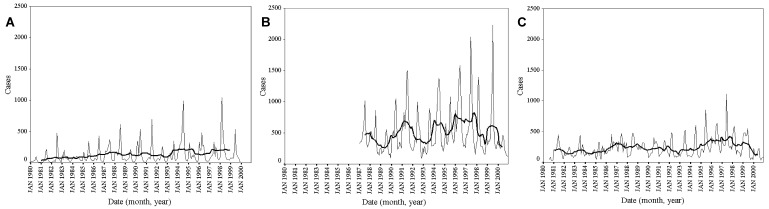
Time series of child admissions for the three study hospitals, Kenya. Time series of child admissions (<15 years of age) for Kilgoris (a), Kisii (b), and Tabaka (c) for 1980–1999, 1987–2000, and 1981–2000 time periods, respectively. The bold line is 25-point (month) moving average of the same data for child admissions.

The trends and interannual variation in pediatric malaria admissions at each facility are shown in Figure 2a–c. These graphs demonstrate clear, substantial between-year variation in child malaria admissions. The 2 years of highest case presentations were 1994 and 1998 for Kilgoris, 1996 and 1997 for Kisii, and 1997 and 1996 for Tabaka (the moving average line in Figure 2a–c clearly shows these years). Although Kisii and Tabaka showed similarities, little coherence occurred in peak years of child admissions between these sites and Kilgoris, despite their close geographic proximity. At each facility, pediatric malaria admissions rose substantially over the period of observation (Table 2). In Kilgoris, deseasonalized child malaria admissions rose from 56 in January 1980 to 200 in December 1999, an increase of 256% over 20 years (p<0.001). Similar trends were observed at Kisii (32% increase from January 1987 through December 1999; p=0.019) and Tabaka (91% increase from January 1980 through December 1999; p<0.001).

**Table 2:** Deseasonalized child admissions at the three study hospitals, Kenya^a^

Site	Data span	Constant	Slope	Change	t value	Significance	Adjusted r^2^
Kilgoris	1980–1999 (n=20)	56.2	7.2	144	6.956	p<0.001	0.165
Kisii	1987–2000 (n=14)	433.8	10.0	140	2.362	p=0.019	0.027
Tabaka	1981–2000 (n=20)	166.4	7.6	152	6.980	p<0.001	0.174

^a^ The data span shows the range of complete years for which data are available (parenthesis indicate the number of observation years). The adjusted r^2^ (sometimes called the coefficient of determination) is goodness-of-fit measure of a linear regression model and varies between 0 and 1. The measure is the proportion of variation in the dependent variable (in this case, malaria admissions) explained by the regression model (in this case, the trend line). The t value is the value of a t-test used to determine if the adjusted r^2^ value is significantly different from 0. The result is shown in the significance column where p values <0.05 are significant.

In parallel with these significant rises in number of cases, estimates of the annual rate of natural population growth in the communities around the hospitals suggest that child populations might have increased by 215%, 49%, and 77% during the same period at Kilgoris, Kisii, and Tabaka, respectively.

## Discussion

We examined longitudinal, age-structured, clinical data on the frequency of admission for severe and complicated *P. falciparum* malaria at the three hospitals located above 1,600 m in the highlands of western Kenya. These data provided an opportunity to explore in more detail several generally accepted positions about the clinical epidemiology of malaria at high altitude in East Africa.

In these time series, the increased malaria admission at each of the three hospitals was concentrated in children <15 years of age (approximately two thirds of all admissions). Given the equivalent sizes of at-risk population below and above 15 years of age (*36*), one must assume that adults have developed a degree of functional immunity to the severe consequences of *P. falciparum* infection. The hypothesis that communities located at high altitude are prone to unstable, infrequent parasite exposure limiting the development of functional immunity before adulthood (*8*,*9*,*30*) is, therefore, not reflected in our data.

Complicated malaria warranting intensive clinical management is a problem every year at each hospital. Previous cross-sectional estimates of the prevalence of *P. falciparum* infection in children from birth to 10 years of age in homesteads in Kisii Central during 1990 suggested infection rates between 4.5% and 13% (*42*). More recently (July 2000), the prevalence of *P. falciparum* infection was 10.3% in children from birth to 9 years of age (HL Guyatt, unpub. data). Neither the clinical epidemiology nor estimates of the prevalence of infection in the community corroborate the view that the high-altitude areas served by the hospitals in our study support unstable transmission. Transmission is better characterized as seasonal and meso-endemic (*43*).

“Highland” malaria is either a new phenomena (*16*–*18*,*23*–*25*,*30*) or a reemergence of a previous prevailing epidemiology (*21*,*44*). Our data confirm significant surges in malaria cases, requiring intensive clinical management during specific years of the 1990s because of substantial overall increases in the number of cases at each hospital. To provide a series of explanations for these increases is tempting, invoking arguments for and against climate change, drug resistance, and land use changes; various authors discuss these arguments elsewhere (*16*–*18*,*20*,*23*–*26*,*30*,*45*–*48*). We emphasize in these arguments, however, the importance of considering population growth as the simplest explanation and note the close correspondence between the percentage increases in the population’s growth rates in the districts served by each facility and the percentage rises in malaria cases. Characteristic of much of sub-Saharan Africa over the last 3 decades, including the highlands of western Kenya, has been a high rate of increase in population size, resulting from high fertility rates and increasing child survival. In the populations served by the hospitals in our study, annual growth rates averaged 3.9%. Under such circumstances, without any change in disease incidence, the increase in disease would be expected to have doubled over approximately an 18-year period. Clearly, without a concomitant investment in essential clinical services, beds, staff, and supporting infrastructure, the changing requirements for clinical management will have been perceived by most district-level public health officials as a crisis.

Defining true epidemics is difficult (*32*). For most public health workers, epidemics represent exacerbations of disease out of proportion to the normal level to which that facility is subject; these increases overwhelm the facility’s ability to cope. Therefore, a slow but pervasive epidemic of clinical malaria may have emerged in the highlands of western Kenya, where lack of investment in the physical capacity to manage an increasing population has resulted inevitably in more malaria cases that require a basic clinical service. In addition to this demographic-to-service determinant, the western highlands are subject to acute seasonal transmission, as evidenced by the temporal distribution of cases (Figure 1a,b). These seasonal peaks in clinical disease exhibit marked between-year variations, and several years exhibit dramatic rises in severe and complicated disease (Figure 2a,b,c). Moreover, years of exceptional cases can be very different between health centers separated by no more than 10 km. With limited resources and bed capacities, these acute rises in disease incidence within a given year will undoubtedly put a considerable strain on any clinical service and represent a crisis (*32*).

We used a crude measure of transmission stability based largely on our understanding of patterns of acquired functional immunity (*8*,*49*). The ACR was derived from hospitalized patients diagnosed with malaria. Many of the cases would not have been confirmed with any degree of reliability through microscopy or careful clinical exclusion of alternative causes for fever (*50*). Our data and approach must therefore be interpreted with this caveat. Nevertheless, in other areas of Kenya where stable transmission is well established (*51*), notably coastal Kwale (ACR = 4,181/6,692 = 0.63 based on admissions data, 1984–1999) and lakeside Homa Bay (ACR = 18,686/35,703 = 0.52 based on admissions data,1982–1999), many more children than adults are admitted to hospital with a malaria diagnosis, resulting in ACRs similar to those described in the highlands (R. Snow, unpub. data). Conversely, in an arid area of northeastern Kenya (Wajir), where a major malaria epidemic occurred in 1998, more adults than children were admitted to the hospital (ACR = 2,704/1,369 = 1.96 based on admissions data, 1988–2000) *(52)*. Despite poor malaria diagnosis in many routine clinical facilities, we believe that the ACR is one possible tool to rapidly assess the extent to which a community has sufficient parasite exposure to invoke some degree of clinical immunity early in childhood. This tool should be explored further within the context of malaria classification for epidemic-prone areas of Africa.

In high-altitude zones of western Kenya, clinical malaria has an acutely seasonal distribution, is comparatively concentrated in the pediatric population, and is a substantial public health problem every year. Occasional, but exceptional, temporal surges of disease occur in some years. We can assume that parasite transmission in this area of Kenya is stable and a degree of functional immunity is acquired during early childhood. Low levels of parasite challenge have been found to be sufficient for early development of functional immunity (*53*). We argue that large parts of the western highlands, located at a similar altitude, have ecologies similar to many other areas with low, stable, but seasonal malaria in Kenya. Treating the highland districts as special cases; demanding intensive investment in early detection, warning, and forecasting systems; and frequent complex-emergency responses by government or nongovernmental organizations (*33*) may not be the most appropriate and cost-effective use of limited resources. Investment in sustainable approaches to vector control (spraying households with residual insecticide), promoting individual protection (insecticide-treated bed nets), and effective case management are perhaps more likely to achieve long-term reductions in disease.
